# Inflammatory Signatures from the Complete Blood Count and In-Hospital Mortality in Acute Myocardial Infarction

**DOI:** 10.3390/jcm15197416

**Published:** 2026-09-24

**Authors:** Alexandra Hurtado-Ortiz, Manuel Santiago Pardo-Muñoz, Andrés Felipe Mora-Salamanca, Juliet López Serrano, Beatriz Mantilla Pérez, Edgar Fabián Manrique-Hernández, Maricel Licht-Ardila, José Federico Saiibi, Ana Milena Cantillo-García

**Affiliations:** 1 Hospital Internacional de Colombia HIC, Fundación Cardiovascular de Colombia FCV, Fundación Universitaria FCV, Km 7 Autopista Bucaramanga—Piedecuesta Valle de Mensulí, Piedecuesta 680001, Colombia; manuelspardo6@gmail.com (M.S.P.-M.); afmorasa@unal.edu.co (A.F.M.-S.); julietvanessa14@gmail.com (J.L.S.); beatrizmantilla@fcv.org (B.M.P.); fabianmh1993@gmail.com (E.F.M.-H.); mlichtar@gmail.com (M.L.-A.); josesaaibi@fcv.org (J.F.S.); acantillo.md@gmail.com (A.M.C.-G.); 2 Postgraduate Department in Infectious Disease, Universidad de Santander, Bucaramanga 680003, Colombia; 3 Escuela de Medicina, Universidad Industrial de Santander, Bucaramanga 680002, Colombia

**Keywords:** myocardial infarction, hospital mortality, blood cell count, inflammation, biomarkers

## Abstract

**Background/Objectives:** Early risk stratification in acute myocardial infarction (AMI) requires accessible and cost-effective tools. Complete blood count-derived inflammatory indices may provide readily available markers of immunothrombotic activity. The objective of this study was to evaluate the association between inflammatory indices derived from the complete blood count at admission and in-hospital mortality among patients with acute myocardial infarction treated at a high-complexity cardiovascular center. **Methods:** We conducted a retrospective cohort analysis of prospectively collected data from adults with type 1 AMI treated at a high-complexity cardiovascular center in Colombia between 2021 and 2025. Complete blood count parameters at admission were used to calculate hematological inflammatory indices. The primary outcome was all-cause in-hospital mortality. Associations were evaluated using logistic regression models. Discrimination was assessed using ROC curves, and restricted cubic splines and sensitivity analyses were used to evaluate nonlinearity and robustness. **Results:** Among 3768 patients, 143 (3.8%) died during hospitalization. Non-survivors had higher NLR, SIRI, SII, and AISI values (all *p* < 0.001). After adjustment, SIRI (OR 1.94, 95% CI 1.62–2.32), NLR (OR 1.89, 95% CI 1.57–2.27), NPR (OR 1.86, 95% CI 1.56–2.22), and AISI (OR 1.83, 95% CI 1.53–2.19) showed the numerically largest adjusted associations with mortality. Discriminatory performance was modest, with the numerically highest AUROC observed for NPR (0.723), followed by SIRI (0.716) and NLR (0.706). **Conclusions:** Inflammatory indices were associated with in-hospital mortality in type 1 AMI, but their discriminatory ability was modest. These indices may serve as complementary markers of inflammatory risk.

## 1. Introduction

Acute myocardial infarction (AMI) remains a major cause of morbidity and mortality and poses a substantial challenge in cardiovascular emergency care [1]. Although high-sensitivity cardiac troponin and established risk scores such as GRACE (Global Registry of Acute Coronary Events) and TIMI (Thrombolysis in Myocardial Infarction) support diagnosis and early risk stratification, their performance depends on the availability and completeness of several clinical and laboratory variables [2,3]. Therefore, accessible and inexpensive biomarkers that may complement current risk assessment remain clinically relevant [4].

Inflammation plays a central role in the pathophysiology and progression of AMI. Myocardial ischemia and necrosis activate the innate immune response through the release of damage-associated molecular patterns [5]. Neutrophil activation contributes to endothelial injury, microvascular obstruction, platelet aggregation, and thrombosis, whereas stress-related lymphopenia and altered monocyte activation may impair the transition from inflammation to tissue repair [3,6]. Metabolic disturbances, including stress hyperglycemia, may further amplify myocardial injury and adverse ventricular remodeling [3,7]. For instance, stress hyperglycemia ratio is associated with a higher incidence of type 4a myocardial infarction in patients with non-ST-segment elevation myocardial infarction (NSTEMI) undergoing percutaneous coronary intervention [8].

These changes can be partially captured through complete blood count-derived inflammatory indices that integrate neutrophils, lymphocytes, monocytes, and platelets. Measures such as the neutrophil-to-lymphocyte ratio (NLR), monocyte-to-lymphocyte ratio (MLR), platelet-to-lymphocyte ratio (PLR), systemic immune-inflammation index (SII), aggregate index of systemic inflammation (AISI), and systemic inflammation response index (SIRI) are inexpensive, reproducible, and widely available [9,10,11]. However, their mathematical overlap and shared cellular components may result in substantial correlation, and their comparative clinical relevance remains uncertain.

Previous studies have associated individual hematological inflammatory indices with adverse outcomes after AMI, but results have varied according to the population, index, follow-up period, and analytical approach [4,9,10,11]. Moreover, much of the available evidence has focused on single indices or selected populations, such as STEMI, patients undergoing percutaneous coronary intervention, or patients with specific comorbidities, limiting direct comparison across markers. Few studies have simultaneously evaluated conventional complete blood count parameters and multiple composite inflammatory indices within the same AMI cohort while accounting for clinical severity, intercorrelation between indices, potential nonlinear associations, and their standalone discriminatory performance [12,13]. Evidence from Latin American cohorts evaluating hematological parameters in AMI remains limited, and additional data from cardiovascular referral centers are needed to determine whether these associations are consistent across different patient profiles and health-system contexts [14]. Therefore, this study aimed to evaluate the association between complete blood count parameters at admission and derived hematological inflammatory indices and all-cause in-hospital mortality among patients with AMI treated at a high-complexity cardiovascular referral center.

## 2. Methods

### 2.1. Study Design and Setting

We conducted a retrospective cohort analysis of prospectively collected data from consecutive adults with type 1 AMI treated between January 2021 and December 2025 at a high-complexity cardiovascular referral center in Colombia. The study was conducted within the Acute Myocardial Infarction Center of Excellence (AMICE), a multidisciplinary program established in 2021 to standardize the diagnosis, treatment, and continuity of care of patients with AMI. The program integrates early clinical assessment, risk stratification, reperfusion when indicated, guideline-directed medical therapy, discharge planning, patient education, cardiac rehabilitation, and longitudinal follow-up.

### 2.2. Study Population

Patients aged 18 years or older with a confirmed diagnosis of type 1 AMI, including ST-segment elevation myocardial infarction (STEMI) and NSTEMI, were eligible (Figure 1). AMI was diagnosed according to contemporary international criteria, requiring a rise and/or fall in cardiac troponin concentrations with at least one value above the 99th percentile upper reference limit, evidence of acute myocardial ischemia, and at least one of the following: symptoms of myocardial ischemia, new ischemic ECG changes, development of pathologic Q waves, imaging evidence of new loss of viable myocardium or new regional wall motion abnormality in a pattern consistent with an ischemic etiology, or identification of a coronary thrombus by angiography or autopsy [15,16]. For patients with more than one AMI hospitalization during the study period, only the first eligible admission was considered the index hospitalization. Subsequent admissions were excluded.

Patients were excluded if they had type 2 myocardial infarction, myocardial injury without evidence of acute atherothrombotic coronary disease, insufficient information to confirm AMI, or no complete blood count available during the index hospitalization. In routine practice, the final diagnosis and differentiation between type 1 AMI, type 2 MI, or non-ischemic myocardial injury were established by the treating cardiology team at discharge following the institutional AMICE protocol. For critically ill or high-risk NSTEMI patients, coronary angiography was the primary tool used to confirm acute atherothrombosis (plaque rupture or occlusion). In patients managed conservatively, type 2 MI or myocardial injury was adjudicated when a documented supply–demand mismatch (e.g., severe sepsis, tachyarrhythmia, severe hemorrhage) or non-ischemic etiology was deemed the primary clinical driver. Consequently, a diagnosis of type 1 AMI in these patients required typical ischemic symptoms or dynamic electrocardiographic changes in the absence of such severe secondary triggers. For analyses of individual inflammatory indices, patients were additionally excluded when any cell count required for its calculation was missing or mathematically undefined.

### 2.3. Data Collection

Clinical information was recorded in a structured REDCap (Research Electronic Data Capture), version 17.3.0, registry [17,18] integrated with the institutional electronic health record system. Trained research personnel reviewed the records, validated clinical information, and resolved inconsistencies using standardized procedures.

Sociodemographic variables included age, sex, educational level, marital status, and area of residence. Clinical variables included smoking status, obesity, hypertension, diabetes mellitus, dyslipidemia, chronic kidney disease, previous coronary artery disease, heart failure, cerebrovascular disease, and chronic obstructive pulmonary disease (COPD).

Variables related to the index event included AMI subtype, Killip class, GRACE and TIMI risk scores, left ventricular ejection fraction, reperfusion strategy, percutaneous coronary intervention, coronary artery bypass grafting, and hospital length of stay.

Laboratory variables included total leukocyte count, red blood cell count, hemoglobin, hematocrit, mean corpuscular volume, mean corpuscular hemoglobin, mean corpuscular hemoglobin concentration, red cell distribution width, platelet count, mean platelet volume, and absolute neutrophil, lymphocyte, monocyte, eosinophil, and basophil counts. Serum creatinine and cardiac troponin were also collected when available.

### 2.4. Hematological Measurements

The first complete blood count obtained during the index hospitalization was used. For all included patients, the complete blood count was obtained within the first 24 h of hospital admission and before coronary revascularization. Complete blood counts were performed as part of routine clinical care using a Sysmex XN-1000 automated hematology analyzer (Sysmex Corporation, Kobe, Japan). Total and differential leukocyte counts and platelet measurements were extracted from the electronic health record. Before analysis, measurement units were standardized and values were assessed for plausibility, timing, and internal consistency.

### 2.5. Hematological Inflammatory Indices

Hematological inflammatory indices were calculated from the absolute blood cell counts obtained from the admission complete blood count (Table 1).

### 2.6. Outcome

The primary outcome was all-cause in-hospital mortality, defined as death from any cause during the index AMI hospitalization. Patients discharged alive were classified as survivors. Vital status was obtained from the AMICE registry and verified against the institutional electronic health record and discharge documentation.

### 2.7. Statistical Analysis

Analyses were conducted using complete cases for the variables included in each model. Consequently, the analytic sample varied slightly across indices and model specifications. The number and percentage of missing observations were summarized for all variables considered in the multivariable analyses and are presented in Supplementary Table S1. No imputation of missing data was performed.

The extent of missing information was described for the principal variables. Continuous variables were assessed for distributional characteristics; normally distributed variables are presented as means with their respective standard deviations, whereas non-normally distributed variables are summarized as medians with interquartile ranges. Categorical variables are reported as frequencies and percentages. Baseline clinical, sociodemographic, and hematological characteristics were compared according to in-hospital survival status using the independent-samples Mann–Whitney *U* test for continuous variables, as appropriate, and χ^2^ test for categorical variables. Correlations among hematological inflammatory indices were examined using Spearman’s rank correlation coefficients. Correlation estimates were displayed in a heat map to describe the degree of overlap among indices.

The association between each complete blood count parameter or hematological inflammatory index and all-cause in-hospital mortality was evaluated using binary logistic regression. Conventional hematological parameters were standardized and modeled per one-standard-deviation increase. The inflammatory indices were naturally log-transformed and subsequently standardized. Their odds ratios therefore represent the change in the odds of in-hospital mortality associated with a one-standard-deviation increase in the log-transformed index.

Crude and multivariable-adjusted odds ratios with 95% confidence intervals were estimated. Each hematological parameter and inflammatory index was evaluated in a separate regression model because several indices share mathematical components and exhibited substantial intercorrelation. The prespecified adjusted model included age, sex, acute myocardial infarction subtype, serum creatinine, log-transformed cardiac troponin, and diabetes mellitus. The primary adjustment set was prespecified to capture demographic characteristics, infarction phenotype, renal function, myocardial injury, and relevant metabolic comorbidity. Acute clinical severity was further evaluated through complementary models incorporating Killip class, GRACE score, and TIMI score, while sensitivity analyses included additional adjustment for PCI and CABG.

The assumption of linearity between each continuous inflammatory index and the log odds of mortality was evaluated using restricted cubic splines applied to the log-transformed index. Four knots were used whenever supported by the observed distribution; three knots were used for indices with insufficient variability for a four-knot specification. Nonlinearity was assessed by jointly testing the spline terms representing deviation from linearity. For indices showing evidence of nonlinearity, adjusted spline plots were generated to characterize the shape of the association. Odds ratios and 95% confidence intervals were estimated using the median value of each log-transformed index as the reference. To reduce instability caused by sparse observations at the distributional extremes, spline curves were restricted to the 5th–95th percentiles. When no evidence of nonlinearity was identified, the association was summarized using the standardized linear term. For indices showing significant nonlinearity, the linear odds ratio was interpreted as an average association across the observed range, and spline findings were considered complementary evidence regarding the shape of the relationship.

The discriminatory ability of each inflammatory index considered individually was evaluated using ROC curves and the area under the curve with 95% confidence intervals. Several sensitivity analyses were conducted. First, the primary models were additionally adjusted for percutaneous coronary intervention and coronary artery bypass grafting. Second, associations were re-estimated using each index on its original scale. Third, the models were repeated using the natural log-transformed but non-standardized index. Robustness was assessed by examining the consistency of the direction and approximate magnitude of associations and the overlap of confidence intervals across model specifications. Analyses were performed using complete cases for the variables included in each model; consequently, sample sizes could vary slightly across analyses. All tests were two-sided, and a *p* value < 0.05 was considered statistically significant. We conducted analyses using Stata version 16 (StataCorp LLC, College Station, TX, USA). Detailed results of the sensitivity analyses are presented in Supplementary Table S1.

## 3. Results

A total of 3768 patients were included in the study. One hundred and forty-three participants (3.8%) experienced in-hospital death (Figure 1). Compared with survivors, patients who died were older (median age, 71.0 vs. 66.0 years; *p* < 0.001) and more frequently presented with STEMI (59.4% vs. 43.8%; *p* < 0.001). No significant differences were observed in terms of sex, marital status, or area of residence. Educational level differed between groups, with a higher proportion of patients without formal education among those who died (17.5% vs. 7.7%; *p* < 0.001). Among prior medical conditions, chronic kidney disease (*p* < 0.001) and peripheral vascular disease (*p* = 0.010) were more frequent in patients who died, whereas dyslipidemia was less common (*p* = 0.012). Other comorbidities did not differ significantly between groups. Length of hospital stay was also longer among patients who died (median, 6.0 vs. 4.0 days; *p* = 0.025). According to the Killip score, clinical severity (Killip III-IV) was greater among those who died in the hospital compared to survivors (*p* < 0.001). Nonetheless, the proportion of missing values was significant (25.4%). In addition, patients who died had statistically significant higher serum creatinine levels, higher cardiac troponin concentrations, and higher GRACE and TIMI scores than survivors (*p* < 0.001) (Table 2).

A total of 4607 patients admitted with suspected acute coronary syndrome between January 2021 and December 2025 were initially screened. After the clinical adjudication process, 617 patients were excluded because they did not meet the criteria for type 1 AMI (this group included patients with type 2 myocardial infarction, non-ischemic myocardial injury, or insufficient clinical information). This left a cohort of 3990 patients with confirmed type 1 acute myocardial infarction. From this group, we further excluded 33 patients without electrocardiographic classification as STEMI or NSTEMI, 119 duplicate records, and 70 patients without a complete blood count at admission, resulting in 3768 patients included in the final analysis. Of these, 3625 survived to hospital discharge and 143 died during hospitalization (Figure 1).

Regarding complete blood count parameters at admission, patients who died had significantly higher white blood cell counts (median, 12.90 vs. 9.85 × 10^3^/µL; *p* < 0.001), absolute neutrophil counts (10.20 vs. 6.91 × 10^3^/µL; *p* < 0.001), and absolute monocyte counts (0.79 vs. 0.68 × 10^3^/µL; *p* < 0.001) compared with survivors. Conversely, red blood cell counts (4.50 vs. 4.76 × 10^3^/µL; *p* < 0.001), hemoglobin levels (13.70 vs. 14.20 g/dL; *p* = 0.007), and absolute lymphocyte counts (1.42 vs. 1.80 × 10^3^/µL; *p* < 0.001) were significantly lower in the non-survivor group. No significant differences were observed between the groups in terms of platelet count (*p* = 0.319), mean platelet volume (*p* = 0.453), mean corpuscular hemoglobin (*p* = 0.129), or absolute basophil count (*p* = 0.678). Furthermore, hematological inflammatory indices were markedly elevated among patients who died during hospitalization. Compared with survivors, non-survivors presented with significantly higher median values for the neutrophil-to-lymphocyte ratio (7.02 vs. 3.78; *p* < 0.001), systemic immune-inflammation index (1625.15 vs. 929.99; *p* < 0.001), aggregate index of systemic inflammation (1384.73 vs. 620.65; *p* < 0.001), and systemic inflammation response index (6.10 vs. 2.50; *p* < 0.001), among other positive indices. In contrast, the monocyte-to-neutrophil ratio was significantly lower in patients who died (0.08 vs. 0.11; *p* < 0.001) (Table 3).

Hematological inflammatory indices showed substantial intercorrelation, reflecting their shared cellular components. The strongest positive correlations were observed between NLR and dNLR (ρ = 0.98), AISI and SIRI (ρ = 0.94), NLR and NLPR (ρ = 0.93), NLR and SII (ρ = 0.93), and dNLR and SII (ρ = 0.91). MNR showed inverse correlations with several neutrophil-based indices, particularly dNLR (ρ = −0.80), NLR (ρ = −0.69), SII (ρ = −0.68), and NLPR (ρ = −0.62) (Figure 2).

The heat map displays pairwise Spearman rank correlation coefficients among complete blood count-derived inflammatory indices. Positive correlations are represented by progressively lighter yellow-green shades, whereas negative correlations are shown in darker blue-purple shades. Values range from −1 to 1, with coefficients closer to either extreme indicating stronger correlations. AISI: aggregate index of systemic inflammation; dNLR: derived neutrophil-to-lymphocyte ratio; MLR: monocyte-to-lymphocyte ratio; MNR: monocyte-to-neutrophil ratio; NLR: neutrophil-to-lymphocyte ratio; NLPR: neutrophil-to-lymphocyte-to-platelet ratio; NPR: neutrophil-to-platelet ratio; PLR: platelet-to-lymphocyte ratio; SII: systemic immune-inflammation index; SIRI: systemic inflammation response index.

In multivariable analyses, higher white blood cell count (adjusted OR 1.58, 95% CI 1.38–1.81), absolute neutrophil count (OR 1.67, 95% CI 1.45–1.92), absolute monocyte count (OR 1.25, 95% CI 1.09–1.43), red cell distribution width (OR 1.33, 95% CI 1.12–1.57), and mean corpuscular volume (OR 1.21, 95% CI 1.02–1.45) were associated with greater odds of in-hospital mortality. In contrast, higher red blood cell count (OR 0.75, 95% CI 0.62–0.89), hemoglobin (OR 0.79, 95% CI 0.66–0.95), and hematocrit (OR 0.79, 95% CI 0.66–0.95) were associated with lower odds. Absolute lymphocyte count showed an inverse association that did not reach conventional statistical significance after adjustment (OR 0.78, 95% CI 0.61–1.00; *p* = 0.052). All hematological inflammatory indices were significantly associated with in-hospital mortality after adjustment, although the direction and magnitude of association varied across indices (Table 4).

Missingness was greatest for TIMI score (42.9%), Killip class (25.4%), and GRACE score (24.3%). Cardiac troponin and serum creatinine were unavailable in 8.5% and 0.7% of patients, respectively. The primary adjusted models included 3435–3436 patients, depending on the hematological parameter or inflammatory index evaluated. The one-patient variation across some models resulted from an unavailable or mathematically undefined inflammatory index value. A detailed description of missing data is provided in Supplementary Table S2.

Among the hematological inflammatory indices, SIRI showed the numerically largest positive association with in-hospital mortality (adjusted OR 1.94, 95% CI 1.62–2.32), followed by NLR (OR 1.89, 95% CI 1.57–2.27), NPR (OR 1.86, 95% CI 1.56–2.22), AISI (OR 1.83, 95% CI 1.53–2.19), NLPR (OR 1.78, 95% CI 1.49–2.12), dNLR (OR 1.78, 95% CI 1.48–2.15), and SII (OR 1.77, 95% CI 1.47–2.13). MLR (OR 1.57, 95% CI 1.31–1.88) and PLR (OR 1.27, 95% CI 1.06–1.52) also showed positive associations, whereas MNR was inversely associated with mortality (OR 0.74, 95% CI 0.63–0.87) (Figure 3).

Across the three multivariable models, NLR, NPR, NLPR, dNLR, SII, AISI, and SIRI remained significantly associated with higher odds of in-hospital mortality. NPR showed the numerically largest association in the GRACE and TIMI models (OR 1.76, 95% CI 1.37–2.25; and OR 1.78, 95% CI 1.27–2.50, respectively), whereas SIRI showed the numerically largest association in the clinical model (OR 1.54, 95% CI 1.21–1.97). MLR remained significant in the clinical and TIMI models but was borderline in the GRACE model. MNR was inversely associated with mortality only in the GRACE model, while PLR was not significantly associated with mortality in any model (Table 5).

Sensitivity analyses were generally consistent with the primary models. Associations remained in the same direction after additional adjustment for PCI and CABG and when indices were modeled using alternative scales. NPR and NLPR showed imprecise estimates when modeled on their original scales, likely reflecting scale-related instability; however, their associations were more stable after log transformation.

Restricted cubic spline analyses showed no evidence of nonlinearity for NLR, NLPR, or dNLR. In contrast, significant nonlinear associations were observed for MLR, NPR, MNR, PLR, AISI, and SIRI, while SII showed borderline evidence of nonlinearity. The adjusted spline curves showed J-shaped patterns for MLR, NPR, PLR, AISI, and SIRI, characterized by lower estimated odds near the lower-to-intermediate portions of their distributions followed by progressively increasing odds at higher values. In contrast, MNR exhibited an inverse nonlinear pattern, with higher estimated odds at low values, decreasing odds across the intermediate range, and a modest increase toward the upper end of its distribution. These associations are presented in Supplementary Figure S1. These findings suggest that, for several indices, the association with in-hospital mortality may not be fully captured by a single linear term.

In discrimination analyses, the individual indices demonstrated modest standalone performance. NPR showed the numerically highest AUROC (0.723), followed by SIRI (0.716), NLR (0.706), and NLPR (0.705). These results indicate that although inflammatory indices were associated with mortality, their ability to discriminate between survivors and non-survivors when used individually was limited. Sensitivity analyses were generally consistent with the primary models. Associations remained in the same direction after additional adjustment for PCI and CABG and when indices were modeled using alternative scales. NPR and NLPR showed imprecise estimates when modeled on their original scales, likely reflecting scale-related instability; however, their associations were more stable after log transformation. Detailed results are presented in Supplementary Table S1.

## 4. Discussion

In this large cohort of patients with type 1 acute myocardial infarction, inflammatory markers derived from the admission complete blood count were consistently associated with all-cause in-hospital mortality. Patients who died had higher leukocyte, neutrophil, and monocyte counts, lower lymphocyte counts, and higher values of most composite inflammatory indices. After multivariable adjustment, SIRI, NLR, NPR, AISI, NLPR, dNLR, and SII showed the largest positive associations, whereas MNR was inversely associated with mortality. However, their standalone discriminatory performance was modest, indicating that these indices should be considered complementary risk markers rather than substitutes for validated clinical scores.

The associations of leukocytosis and neutrophilia with mortality are consistent with previous evidence in acute coronary syndromes. In the TACTICS–TIMI 18 substudy, higher baseline leukocyte counts were associated with more extensive coronary disease, impaired myocardial perfusion, and increased mortality [13]. Similar findings have been reported in STEMI, where neutrophilia has been related to greater infarct size, microvascular obstruction, heart failure, and death. Our study adds evidence to a contemporary population including both STEMI and NSTEMI and shows that these associations persist after accounting for major clinical risk factors.

These findings are biologically plausible because neutrophils play a central role in ischemic and reperfusion injury. Myocardial necrosis promotes neutrophil mobilization and recruitment to injured tissue, where activated cells release proteolytic enzymes, reactive oxygen species, cytokines, and neutrophil extracellular traps. Although this response contributes to debris clearance and tissue repair, excessive activation may aggravate endothelial dysfunction, thrombosis, microvascular obstruction, and myocardial injury. Conversely, lymphopenia may reflect stress-induced neuroendocrine activation, immune dysregulation, comorbidity, or hemodynamic severity. Because lymphocytes form the denominator of several indices, their reduction amplifies NLR, MLR, PLR, NLPR, SII, AISI, and SIRI.

NLR was one of the most consistent markers across the different model specifications. This agrees with the findings of Tamhane et al. in 2833 patients with acute coronary syndromes [19], subsequent studies in STEMI [20], and meta-analytic evidence showing that elevated NLR is associated with mortality and major adverse cardiovascular events after myocardial infarction [21]. Its prognostic relevance may arise from simultaneously capturing neutrophil-mediated innate activation and relative lymphocyte depletion. Nevertheless, NLR is nonspecific and can be influenced by infection, corticosteroid use, smoking, malignancy, chronic inflammation, physiological stress, and the timing of blood collection. Moreover, recent evidence in STEMI patients undergoing primary PCI has shown that the Advanced Lung Cancer Inflammation Index (ALI), a composite inflammatory–nutritional marker, has greater prognostic value than the NLR. These findings suggest that readily available hematological indices may enhance their predictive performance when combined with other easily accessible clinical variables, such as nutritional status, in the setting of acute cardiovascular care [22].

SIRI presented the numerically largest adjusted association in the primary analysis and remained significant across the clinical, GRACE, and TIMI models. By combining neutrophils, monocytes, and lymphocytes, SIRI may reflect innate immune activation, monocyte recruitment, and relative adaptive immune suppression. Previous studies have also associated elevated SIRI with adverse outcomes in acute coronary syndrome and STEMI [23,24]. SII and AISI were similarly associated with mortality, in agreement with reports linking these indices to adverse cardiovascular events and survival after PCI and AMI [24,25,26].

However, the more complex indices did not clearly outperform simpler markers. NLR was almost perfectly correlated with dNLR and strongly correlated with NLPR and SII, while AISI and SIRI were also highly correlated. These indices should therefore be understood as alternative mathematical representations of a common inflammatory phenotype dominated by neutrophilia, lymphopenia, and, to a lesser extent, monocytosis and platelet variation. This overlap supports evaluating them in separate models and limits claims of biological independence, or that any single index represents a distinct biological pathway or is definitively superior.

NPR produced the numerically highest AUROC and remained associated with mortality across the adjusted analyses. Nevertheless, NPR has been less extensively studied than NLR, PLR, SII, or SIRI, and its estimates on the original scale were imprecise. Because platelet count alone was not associated with mortality, the NPR result may have been driven mainly by neutrophil elevation rather than an independent platelet-related mechanism. In contrast, PLR showed weaker and less consistent results: although significant in the primary model, it lost significance after adjustment for clinical severity and established risk scores. Previous meta-analyses have associated PLR with adverse outcomes after acute coronary syndromes [27,28], but their findings have been heterogeneous, possibly because of differences in populations, cutoff values, treatment strategies, follow-up, and covariate adjustment.

MNR was inversely associated with mortality in the primary model but was not consistently significant in the more comprehensive models. This inverse association is mathematically compatible with the increased neutrophil burden among patients who died and should not be interpreted as evidence that monocytes are protective. Monocytes participate in both inflammatory injury and myocardial repair, and their prognostic role may depend on the timing and relative kinetics of leukocyte mobilization.

Among conventional hematological parameters, higher RDW remained independently associated with mortality, in agreement with previous meta-analytic evidence in acute coronary syndrome [29]. RDW may integrate inflammation, oxidative stress, impaired erythropoiesis, renal dysfunction, nutritional deficiencies, and chronic disease burden. Lower red blood cell count, hemoglobin, and hematocrit were also associated with mortality, possibly reflecting reduced oxygen-carrying capacity or greater underlying comorbidity. However, these variables are strongly interrelated and should not be interpreted as independent biological effects.

Despite statistically significant associations, the AUROC values of the individual indices were modest. Thus, their distributions overlapped substantially between survivors and patients who died, limiting their use as standalone classifiers. Their main potential advantage is that they are inexpensive, rapidly available, and routinely measured at admission. However, the present analysis does not establish that they improve prediction beyond GRACE, TIMI, or other validated models. Such a conclusion would require direct comparison of models with and without each index, together with assessment of calibration, internal validation, and clinical net benefit.

The nonlinear relationships observed for MLR, NPR, MNR, PLR, AISI, and SIRI also indicate that a single linear odds ratio or fixed cutoff may oversimplify their associations with mortality. This may partly explain the heterogeneous thresholds reported in previous studies. Continuous modeling using logarithmic transformations and flexible functions may provide a more reliable representation than data-derived cutoffs, which frequently have limited transportability.

This study has several strengths, including its large contemporary cohort, the inclusion of both STEMI and NSTEMI, and the simultaneous evaluation of conventional blood count parameters and multiple inflammatory indices. The analyses also addressed intercorrelation, nonlinear associations, discrimination, and robustness across alternative adjustment models. Nevertheless, several limitations should be acknowledged. First, the retrospective analysis of registry data precludes causal inference and remains susceptible to residual confounding. Information on concurrent infections, chronic inflammatory or hematological diseases, active malignancy, and corticosteroid exposure was not systematically captured. These non-cardiac determinants of blood count abnormalities may therefore have contributed to unmeasured confounding. Future prospective studies should systematically document these conditions to better distinguish the inflammatory response associated with myocardial ischemia from other systemic triggers.

Although complementary models incorporated Killip class, GRACE score, and TIMI score, and sensitivity analyses additionally adjusted for PCI and CABG, acute clinical severity may not have been fully captured. LVEF was not included in the multivariable models, and direct indicators of hemodynamic instability, including cardiogenic shock, vasopressor requirement, mechanical circulatory support, and sustained hypotension, were not modeled separately. Moreover, adjustment for PCI and CABG did not encompass all aspects of reperfusion management, such as fibrinolysis, absence of reperfusion, and treatment timing. Residual confounding related to ventricular dysfunction, hemodynamic compromise, infarct severity, and treatment selection therefore cannot be excluded.

All complete blood counts were obtained within the first 24 h of admission and before coronary revascularization; however, the exact timing relative to symptom onset was not uniformly available. This is relevant because inflammatory indices may change dynamically during the acute course of myocardial infarction. In addition, the complete-case approach may have introduced selection bias, particularly in models incorporating Killip class, GRACE score, and TIMI score, for which substantial proportions of observations were missing. The single-center design and the potential influence of local care pathways may also limit external generalizability, and the findings should therefore be considered primarily hypothesis-generating. Furthermore, evaluating multiple correlated indices increases the possibility of chance findings. Finally, discrimination was assessed for each index individually, without formally evaluating incremental predictive value beyond established risk scores or standard clinical models through changes in C-statistics, calibration, reclassification measures, or decision-curve analysis. Accordingly, these findings should not be interpreted as evidence that the inflammatory indices improve clinical risk prediction.

## 5. Conclusions

Admission complete blood count-derived inflammatory indices were associated with all-cause in-hospital mortality in patients with type 1 AMI. Neutrophil-dominant indices, particularly SIRI, NLR, NPR, AISI, NLPR, dNLR, and SII, showed the most consistent associations, whereas PLR and MNR were less robust after adjustment for clinical severity. Their high intercorrelation suggests that these indices reflect a shared inflammatory phenotype. Although they were associated with in-hospital mortality, their standalone discriminatory ability was modest, and the present study did not demonstrate incremental predictive value beyond established clinical assessment. Accordingly, these indices should be interpreted as exploratory markers of inflammatory risk rather than as validated tools for improving clinical risk prediction. Rather than supporting immediate broad clinical implementation, our findings serve primarily as a robust local validation of previously described indices within a Latin American healthcare context. External validation across diverse populations is needed to determine whether these selected indices provide meaningful prognostic information beyond established tools like the GRACE and TIMI scores.

## Figures and Tables

**Figure 1:**
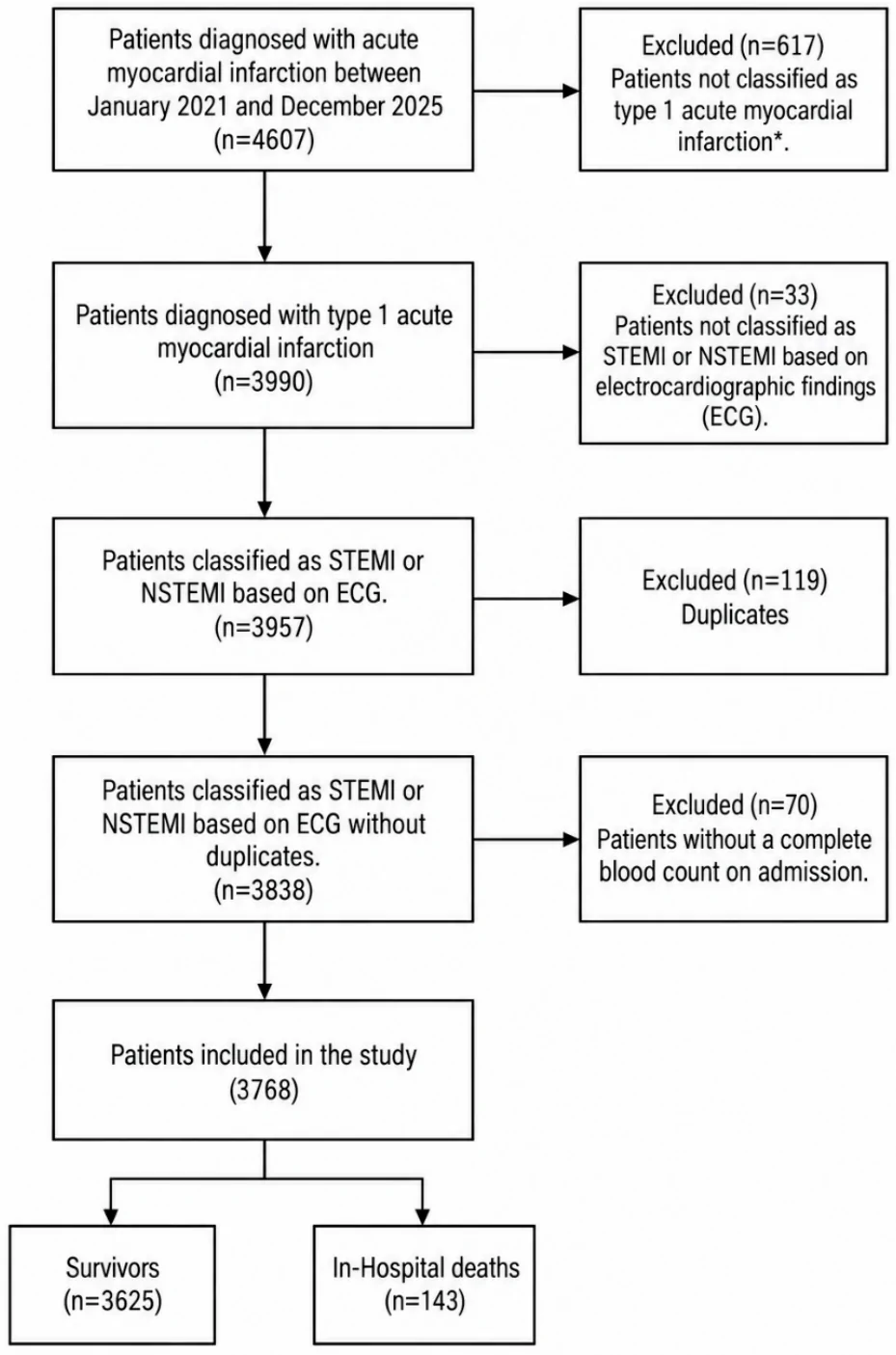
Study flowchart of included participants. * Patients not classified as type 1 myocardial infarction included patients with type 2 myocardial infarction, non-ischemic injury, or medical records with insufficient information. ECG: electrocardiogram; NSTEMI: non-ST-segment elevation myocardial infarction; STEMI: ST-segment elevation myocardial infarction.

**Figure 2:**
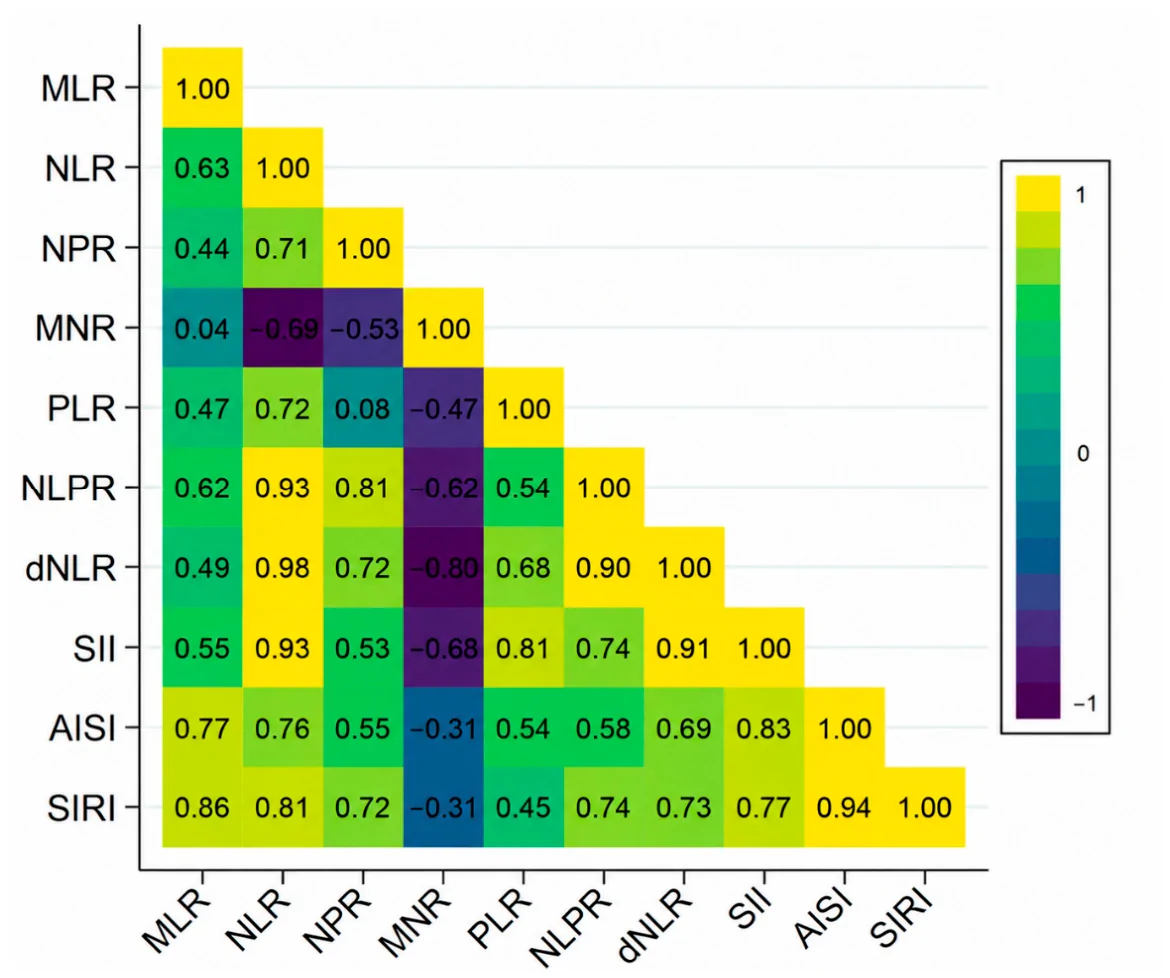
Spearman Correlation Matrix of Hematological Inflammatory Indices.

**Figure 3:**
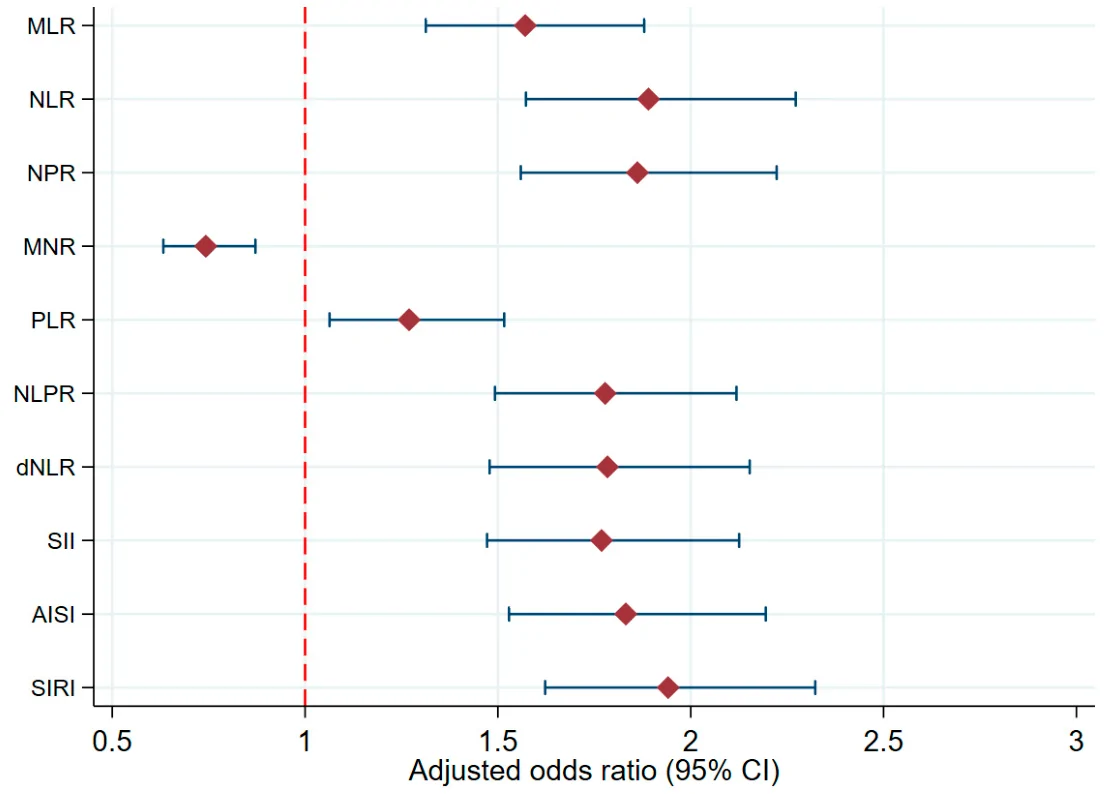
Adjusted associations between hematological inflammatory indices and in-hospital mortality. Diamonds represent adjusted odds ratios and horizontal lines indicate 95% confidence intervals. The red dashed vertical line indicates the null value of association (adjusted odds ratio = 1).

**Table 1:** Hematological inflammatory index definitions.

Index	Definition
Monocyte-to-lymphocyte ratio	MLR = monocyte count/lymphocyte count
Neutrophil-to-lymphocyte ratio	NLR = neutrophil count/lymphocyte count
Neutrophil-to-platelet ratio	NPR = neutrophil count/platelet count
Monocyte-to-neutrophil ratio	MNR = monocyte count/neutrophil count
Platelet-to-lymphocyte ratio	PLR = platelet count/lymphocyte count
Neutrophil-to-lymphocyte-to-platelet ratio	NLPR = neutrophil count/(lymphocyte count × platelet count)
Derived neutrophil-to-lymphocyte ratio	dNLR = neutrophil count/(total leukocyte count − neutrophil count)
Systemic immune-inflammation index	SII = (platelet count × neutrophil count)/lymphocyte count
Aggregate index of systemic inflammation	AISI = (neutrophil count × monocyte count × platelet count)/lymphocyte count
Systemic inflammation response index	SIRI = (neutrophil count × monocyte count)/lymphocyte count

Numerators and denominators were expressed in cells × 10^3^/µL. Values were considered mathematically undefined and treated as missing when a denominator was equal to zero. Implausible extreme values were verified against the original laboratory report and retained when confirmed as clinically plausible.

**Table 2:** Baseline Sociodemographic and Clinical Characteristics According to In-Hospital Mortality.

Characteristic	Total (*n* = 3768)	Alive (*n* = 3625)	In-Hospital Death (*n* = 143)	*p* Value
Age, years, median (IQR)	66.0 (58.0–74.0)	66.0 (58.0–74.0)	71.0 (63.0–77.0)	<0.001
Sex, *n* (%)				0.666
Male	2721 (72.2%)	2620 (72.3%)	101 (70.6%)	
Female	1047 (27.8%)	1005 (27.7%)	42 (29.4%)	
Marital status				0.390
Without permanent partner	1277 (33.9%)	1223 (33.7%)	54 (37.8%)	
With permanent partner	2376 (63.1%)	2293 (63.3%)	83 (58.0%)	
Missing	115 (3.1%)	109 (3.0%)	6 (4.2%)	
Area of residence				0.950
Urban	3069 (81.4%)	2952 (81.4%)	117 (81.8%)	
Rural	699 (18.6%)	673 (18.6%)	26 (18.2%)	
Educational level				<0.001
No formal education	305 (8.1%)	280 (7.7%)	25 (17.5%)	
Primary education	2072 (55.0%)	1999 (55.1%)	73 (51.0%)	
Secondary education	811 (21.5%)	788 (21.7%)	23 (16.1%)	
Technical or technological education	136 (3.6%)	130 (3.6%)	6 (4.2%)	
University or postgraduate education	279 (7.4%)	264 (7.3%)	15 (10.5%)	
No information	165 (4.4%)	164 (4.5%)	1 (0.7%)	
Medical History				
Hypertension	2420 (64.2%)	2336 (64.4%)	84 (58.7%)	0.160
Diabetes mellitus	1212 (32.2%)	1156 (31.9%)	56 (39.2%)	0.068
Dyslipidemia	1176 (31.2%)	1145 (31.6%)	31 (21.7%)	0.012
History of CAD	232 (6.2%)	227 (6.3%)	5 (3.5%)	0.180
Chronic kidney disease	202 (5.4%)	181 (5.0%)	21 (14.7%)	<0.001
COPD	121 (3.2%)	114 (3.1%)	7 (4.9%)	0.240
Congestive heart failure	62 (1.6%)	58 (1.6%)	4 (2.8%)	0.270
Peripheral vascular disease	32 (0.8%)	28 (0.8%)	4 (2.8%)	0.010
Stroke	14 (0.4%)	13 (0.4%)	1 (0.7%)	0.510
Current smoking	477 (12.7%)	462 (12.7%)	15 (10.5%)	0.430
Alcohol consumption	428 (11.4%)	417 (11.5%)	11 (7.7%)	0.160
Obesity	1097 (29.1%)	1062 (29.3%)	35 (24.5%)	0.210
Type of AMI				<0.001
STEMI	1671 (44.3%)	1586 (43.8%)	85 (59.4%)	
NSTEMI	2097 (55.7%)	2039 (56.2%)	58 (40.6%)	
LVEF, %, median (IQR)	50 (40-55)	50 (40-55)	35 (25-45)	<0.001
Type of intervention				
PCI	2786 (74.0%)	2681 (74.0%)	105 (73.4%)	0.880
CABG	784 (20.8%)	772 (21.3%)	12 (8.4%)	<0.001
Medical Treatment	464 (12.3%)	432 (11.9%)	32 (22.4%)	<0.001
Killip class				<0.001
Killip I	2215 (58.8%)	2186 (60.3%)	29 (20.3%)	
Killip II	326 (8.7%)	310 (8.6%)	16 (11.2%)	
Killip III	183 (4.9%)	161 (4.4%)	22 (15.4%)	
Killip IV	86 (2.3%)	57 (1.6%)	29 (20.3%)	
Missing	958 (25.4%)	911 (25.1%)	47 (32.9%)	
Serum creatinine, mg/dL	0.94 (0.8–1.2)	0.93 (0.8–1.2)	1.25 (0.9–1.9)	<0.001
Cardiac troponin	1071.10 (230.7–6009.4)	1020.00 (222.0–5848.0)	3621.00 (532.80–19,018.6)	<0.001
GRACE score	118.0 (98.0–138.0)	117.0 (97.0–136.0)	152.5 (124.0–193.0)	<0.001
TIMI score	3.0 (2.0–4.0)	3.0 (2.0–4.0)	4.0 (3.0–5.0)	<0.001
Length of hospital stay, days	4.0 (2.0–10.0)	4.0 (2.0–10.0)	6.0 (2.0–12.0)	0.025

Data are presented as median (interquartile range [IQR]) for continuous variables and as number (percentage) for categorical variables. Comparisons between survivors and patients who died during hospitalization were performed using the Mann–Whitney U test for continuous variables and the chi-square test or Fisher’s exact test for categorical variables, as appropriate. Percentages were calculated using the total number of patients within each comparison group as the denominator. AMI: acute myocardial infarction; CABG: coronary artery bypass grafting; COPD: chronic obstructive pulmonary disease; GRACE: Global Registry of Acute Coronary Events; NSTEMI: non-ST-segment elevation myocardial infarction; PCI: percutaneous coronary intervention; STEMI: ST-segment elevation myocardial infarction; TIMI: Thrombolysis in Myocardial Infarction; CAD: coronary artery disease. PCI and CABG were not mutually exclusive because some patients underwent both procedures during the index hospitalization; therefore, percentages across revascularization procedures may exceed 100%. Medical treatment was defined as the absence of both PCI and CABG during the index hospitalization.

**Table 3:** Baseline Complete Blood Count Parameters and Hematological Inflammatory Indices According to In-Hospital Mortality.

Characteristic	Alive (*n* = 3625) Median (IQR)	In-Hospital Death (*n* = 143) Median (IQR)	*p* Value
White blood cell count (×10^3^/µL)	9.85 (7.88–12.20)	12.90 (10.01–17.27)	<0.001
Red blood cell count (×10^6^/µL)	4.76 (4.31–5.18)	4.50 (4.07–5.00)	<0.001
Hemoglobin (g/dL)	14.20 (12.80–15.30)	13.70 (12.00–15.20)	0.007
Hematocrit (%)	42 (38–46)	41 (37–45)	0.007
Mean corpuscular volume [MCV] (fL)	88.50 (85.60–91.60)	89.90 (86.30–93.20)	0.002
Mean corpuscular hemoglobin [MCH] (pg)	29.80 (28.70–30.80)	30.10 (28.90–31.10)	0.129
Mean corpuscular hemoglobin concentration [MCHC] (g/dL)	33.60 (32.70–34.50)	33.50 (32.10–34.50)	0.537
Platelet count (×10^3^/µL)	248.00 (206.00–293.0)	246.00 (193.00–293.00)	0.319
Red cell distribution width [RDW] (%)	12.70 (12.00–13.40)	13.00 (12.40–14.00)	<0.001
Mean platelet volume [MPV] (fL)	9.79 (8.90–10.47)	9.60 (8.61–10.50)	0.453
Absolute neutrophil count (×10^3^/µL)	6.91 (5.02–9.41)	10.20 (7.51–14.70)	<0.001
Absolute lymphocyte count (×10^3^/µL)	1.80 (1.26–2.41)	1.42 (0.99–2.01)	<0.001
Absolute monocyte count (×10^3^/µL)	0.68 (0.52–0.89)	0.79 (0.57–1.13)	<0.001
Absolute eosinophil count (×10^3^/µL)	0.07 (0.02–0.17)	0.04 (0.01–0.14)	0.005
Absolute basophil count (×10^3^/µL)	0.04 (0.03–0.06)	0.04 (0.03–0.07)	0.678
Neutrophils (%)	72 (62–81)	77 (68–85)	<0.001
Lymphocytes (%)	19 (12–26)	14 (09–23)	<0.001
Monocytes (%)	7 (6–9)	7 (5–9)	0.020
Eosinophils (%)	1 (0–2)	0 (0–1)	0.001
Basophils (%)	0.4 (0.1–0.9)	0.2 (0.0–0.6)	0.008
MLR	0.37 (0.27–0.53)	0.57 (0.36–0.90)	<0.001
NLR	3.78 (2.33–6.61)	7.02 (4.24–12.23)	<0.001
NPR	0.03 (0.02–0.04)	0.04 (0.03–0.06)	<0.001
MNR	0.11 (0.07–0.14)	0.08 (0.05–0.11)	<0.001
PLR	137.73 (100.36–196.52)	161.24 (111.95–226.04)	0.001
NLPR	0.02 (0.01–0.03)	0.03 (0.02–0.05)	<0.001
dNLR	2.43 (1.62–4.03)	4.28 (2.59–6.36)	<0.001
SII	929.99 (353.98–1657.36)	1625.15 (929.62–2844.00)	<0.001
AISI	620.65 (353.98–1147.16)	1384.73 (591.87–2759.36)	<0.001
SIRI	2.50 (1.49–4.50)	6.10 (2.77–11.41)	<0.001

Data are presented as median (interquartile range [IQR]). Comparisons between survivors and patients who died during hospitalization were performed using the Mann–Whitney U test. AISI: aggregate index of systemic inflammation; dNLR: derived neutrophil-to-lymphocyte ratio; MCH: mean corpuscular hemoglobin; MCHC: mean corpuscular hemoglobin concentration; MCV: mean corpuscular volume; MLR: monocyte-to-lymphocyte ratio; MNR: monocyte-to-neutrophil ratio; MPV: mean platelet volume; NLPR: neutrophil-to-lymphocyte-to-platelet ratio; NLR: neutrophil-to-lymphocyte ratio; NPR: neutrophil-to-platelet ratio; PLR: platelet-to-lymphocyte ratio; RDW: red cell distribution width; SII: systemic immune-inflammation index; SIRI: systemic inflammation response index.

**Table 4:** Crude and Adjusted Associations of Complete Blood Count Parameters and Hematological Inflammatory Indices With In-Hospital Mortality.

Parameter	Crude OR (95% CI)	*p* Value	*n*	Adjusted OR (95% CI)	*p* Value	*n*
White blood cell count	1.66 (1.47–1.88)	<0.001	3768	1.58 (1.38–1.81)	<0.001	3436
Red blood cell count	0.73 (0.62–0.86)	<0.001	3768	0.75 (0.62–0.89)	0.001	3436
Hemoglobin	0.79 (0.68–0.93)	0.004	3768	0.79 (0.66–0.95)	0.012	3436
Hematocrit	0.80 (0.68–0.94)	0.006	3768	0.79 (0.66–0.95)	0.011	3436
Mean corpuscular volume	1.29 (1.10–1.51)	0.002	3768	1.21 (1.02–1.45)	0.033	3436
Mean corpuscular hemoglobin	1.16 (0.98–1.37)	0.082	3768	1.13 (0.94–1.36)	0.184	3436
Mean corpuscular hemoglobin concentration	0.96 (0.81–1.13)	0.592	3768	1.00 (0.84–1.20)	0.959	3436
Platelet count	0.92 (0.78–1.10)	0.384	3768	1.03 (0.86–1.23)	0.784	3436
Red cell distribution width	1.32 (1.13–1.54)	<0.001	3768	1.33 (1.12–1.57)	0.001	3436
Mean platelet volume	0.92 (0.78–1.09)	0.344	3767	1.04 (0.87–1.25)	0.650	3436
Absolute neutrophil count	1.75 (1.54–1.98)	<0.001	3768	1.67 (1.45–1.92)	<0.001	3436
Absolute lymphocyte count	0.73 (0.58–0.92)	0.009	3768	0.78 (0.61–1.00)	0.052	3436
Absolute monocyte count	1.38 (1.19–1.59)	<0.001	3768	1.25 (1.09–1.43)	0.001	3436
Absolute basophil count	1.16 (1.02–1.33)	0.024	3768	1.11 (0.94–1.30)	0.217	3436
MLR	1.80 (1.53–2.12)	<0.001	3767	1.57 (1.31–1.88)	<0.001	3435
NLR	2.05 (1.74–2.41)	<0.001	3767	1.89 (1.57–2.27)	<0.001	3435
NPR	2.07 (1.76–2.43)	<0.001	3768	1.86 (1.56–2.22)	<0.001	3436
MNR	0.74 (0.64–0.85)	<0.001	3768	0.74 (0.63–0.87)	<0.001	3436
PLR	1.30 (1.11–1.53)	0.001	3767	1.27 (1.06–1.52)	0.008	3435
NLPR	1.99 (1.70–2.32)	<0.001	3767	1.78 (1.49–2.12)	<0.001	3435
dNLR	1.87 (1.59–2.21)	<0.001	3768	1.78 (1.48–2.15)	<0.001	3436
SII	1.85 (1.58–2.18)	<0.001	3767	1.77 (1.47–2.13)	<0.001	3435
AISI	1.99 (1.70–2.33)	<0.001	3767	1.83 (1.53–2.19)	<0.001	3435
SIRI	2.15 (1.83–2.52)	<0.001	3767	1.94 (1.62–2.32)	<0.001	3435

Odds ratios for conventional complete blood count parameters are reported per one-standard-deviation increase. Hematological inflammatory indices were natural log-transformed and standardized; their odds ratios therefore represent the change in the odds of in-hospital mortality per one-standard-deviation increase in the log-transformed index. Adjusted models included age, sex, acute myocardial infarction subtype, serum creatinine, log-transformed cardiac troponin, and diabetes mellitus. Each parameter or index was evaluated in a separate logistic regression model. Sample sizes varied because of missing data. The absolute eosinophil count was excluded because its regression estimates were numerically unstable and produced a noninformative confidence interval. AISI: aggregate index of systemic inflammation; CI: confidence interval; dNLR: derived neutrophil-to-lymphocyte ratio; MLR: monocyte-to-lymphocyte ratio; MNR: monocyte-to-neutrophil ratio; NLR: neutrophil-to-lymphocyte ratio; NLPR: neutrophil-to-lymphocyte-to-platelet ratio; NPR: neutrophil-to-platelet ratio; OR: odds ratio; PLR: platelet-to-lymphocyte ratio; SII: systemic immune-inflammation index; SIRI: systemic inflammation response index.

**Table 5:** Adjusted Associations Between Hematological Inflammatory Indices and In-Hospital Mortality Across Clinical, GRACE, and TIMI Models.

Index	Clinical Model OR (95% CI)	*p* Value	GRACE Model OR (95% CI)	*p* Value	TIMI Model OR (95% CI)	*p* Value
MLR	1.29 (1.03–1.61)	0.027	1.23 (0.99–1.53)	0.056	1.39 (1.03–1.88)	0.033
NLR	1.47 (1.14–1.88)	0.003	1.52 (1.21–1.92)	<0.001	1.48 (1.07–2.05)	0.018
NPR	1.46 (1.14–1.86)	0.002	1.76 (1.37–2.25)	<0.001	1.78 (1.27–2.50)	0.001
MNR	0.87 (0.69–1.09)	0.217	0.78 (0.63–0.96)	0.020	0.92 (0.68–1.24)	0.573
PLR	1.11 (0.89–1.39)	0.333	1.12 (0.90–1.40)	0.325	1.06 (0.79–1.44)	0.691
NLPR	1.38 (1.08–1.75)	0.009	1.50 (1.19–1.90)	0.001	1.45 (1.06–2.00)	0.022
dNLR	1.39 (1.08–1.80)	0.011	1.51 (1.19–1.91)	0.001	1.39 (1.00–1.94)	0.048
SII	1.41 (1.10–1.80)	0.006	1.46 (1.15–1.84)	0.002	1.41 (1.02–1.94)	0.037
AISI	1.49 (1.17–1.90)	0.001	1.46 (1.16–1.84)	0.001	1.62 (1.17–2.24)	0.004
SIRI	1.54 (1.21–1.97)	0.001	1.53 (1.21–1.92)	<0.001	1.72 (1.23–2.39)	0.001

Odds ratios represent the association with in-hospital mortality per one-standard-deviation increase in the natural log-transformed index. The clinical model included age, sex, educational level, diabetes mellitus, dyslipidemia, serum creatinine, acute myocardial infarction subtype, log-transformed troponin, and Killip class. The GRACE model included sex, educational level, diabetes mellitus, dyslipidemia, acute myocardial infarction subtype, log-transformed troponin, and GRACE score. The TIMI model included the clinical-model covariates with additional adjustment for the TIMI risk score. Sample sizes varied across models because of missing data in covariates, particularly Killip class and risk scores. AISI: aggregate index of systemic inflammation; CI: confidence interval; dNLR: derived neutrophil-to-lymphocyte ratio; MLR: monocyte-to-lymphocyte ratio; MNR: monocyte-to-neutrophil ratio; NLR: neutrophil-to-lymphocyte ratio; NLPR: neutrophil-to-lymphocyte-to-platelet ratio; NPR: neutrophil-to-platelet ratio; OR: odds ratio; PLR: platelet-to-lymphocyte ratio; SII: systemic immune-inflammation index; SIRI: systemic inflammation response index.

## Data Availability

The datasets presented in this article are not readily available because of institutional privacy restrictions regarding patient data. Requests to access the datasets should be directed to the corresponding author.

## Supplementary Materials

The following supporting information can be downloaded at https://www.mdpi.com/article/10.3390/jcm15197416/s1, Table S1: Sensitivity analysis of the association between hematological inflammatory indices and in-hospital mortality. Table S2: Missing data for variables considered in the multivariable analyses. Figure S1. Restricted cubic spline associations between selected inflammatory indices and in-hospital mortality.
