# Supplementary material for: Inflammatory Signatures from the Complete Blood Count and In-Hospital Mortality in Acute Myocardial Infarction
Source: J Clin Med. 2026 Sep 24;15(19):7416. doi: 10.3390/jcm15197416 (PMC13666292; doi:10.3390/jcm15197416)
Supplement: Supplementary file 1 [file jcm-15-07416-s001.zip › jcm-4454510-supplementary.pdf]

Table S1. Sensitivity Analysis of the Association Between Hematological Inflammatory Indices and In-Hospital Mortality.

Index	Main model OR (95% CI)	Main + PCI/CABG OR (95% CI)	Original index OR (95% CI)	Log-transformed index OR (95% CI)
MLR	1.57 (1.31–1.88)	1.50 (1.25–1.80)	2.74 (1.88–3.99)	2.21 (1.61–3.02)
NLR	1.89 (1.57–2.27)	1.81 (1.50–2.18)	1.07 (1.05–1.09)	2.36 (1.84–3.03)
NPR	1.86 (1.56–2.22)	1.79 (1.50–2.15)	3.89 (0.75–20.31)	3.41 (2.41–4.85)
MNR	0.74 (0.63–0.87)	0.75 (0.64–0.88)	0.005 (0.000–0.281)	0.60 (0.46–0.79)
PLR	1.27 (1.06–1.52)	1.23 (1.03–1.47)	1.002 (1.001–1.003)	1.56 (1.12–2.18)
NLPR	1.78 (1.49–2.12)	1.70 (1.42–2.03)	11.24 (0.84–151.23)	2.06 (1.65–2.57)
dNLR	1.78 (1.48–2.15)	1.72 (1.42–2.08)	1.13 (1.07–1.18)	2.41 (1.81–3.20)
SII	1.77 (1.47–2.13)	1.70 (1.42–2.05)	1.000 (1.000–1.000)	2.04 (1.62–2.57)
AISI	1.83 (1.53–2.20)	1.77 (1.47–2.12)	1.000 (1.000–1.000)	1.92 (1.58–2.33)
SIRI	1.94 (1.62–2.32)	1.86 (1.55–2.23)	1.04 (1.02–1.06)	2.14 (1.74–2.63)

The main model included age, sex, acute myocardial infarction subtype, serum creatinine, log-transformed cardiac troponin, and diabetes mellitus. The PCI/CABG model additionally included percutaneous coronary intervention and coronary artery bypass grafting. In the main and PCI/CABG models, inflammatory indices were natural log-transformed and standardized; odds ratios represent the association with in-hospital mortality per one-standard-deviation increase in the log-transformed index. Models using the original and non-standardized log-transformed indices were performed as sensitivity analyses. Odds ratios across these specifications are not directly comparable because they are expressed on different scales. AISI: aggregate index of systemic inflammation; CABG: coronary artery bypass grafting; CI: confidence interval; dNLR: derived neutrophil-to-lymphocyte ratio; MLR: monocyte-to-lymphocyte ratio; MNR: monocyte-to-neutrophil ratio; NLR: neutrophil-to-lymphocyte ratio; NLPR: neutrophil-to-lymphocyte-to-platelet ratio; NPR: neutrophil-to-platelet ratio; OR: odds ratio; PCI: percutaneous coronary intervention; PLR: platelet-to-lymphocyte ratio; SII: systemic immune-inflammation index; SIRI: systemic inflammation response index.

Supplementary Table S2. Missing data for variables considered in the multivariable analyses

Variable	Available, n (%)	Missing, n (%)
Age	3,768 (100.0)	0 (0.0)
Sex	3,768 (100.0)	0 (0.0)
Diabetes mellitus	3,768 (100.0)	0 (0.0)
Dyslipidemia	3,768 (100.0)	0 (0.0)
AMI subtype	3,768 (100.0)	0 (0.0)
Educational level	3,603 (95.6)	165 (4.4)
Serum creatinine	3,743 (99.3)	25 (0.7)
Cardiac troponin	3,448 (91.5)	320 (8.5)
Killip class	2,810 (74.6)	958 (25.4)
GRACE score	2,853 (75.7)	915 (24.3)
TIMI score	2,153 (57.1)	1,615 (42.9)

Data are presented as the number and percentage of the total study population (N = 3,768). Percentages may not total 100% because of rounding. Multivariable analyses were performed using complete cases for the variables included in each model; no missing-data imputation was conducted. AMI, acute myocardial infarction; GRACE, Global Registry of Acute Coronary Events; TIMI, Thrombolysis in Myocardial Infarction.

Figure S1. Restricted cubic spline associations between selected inflammatory indices and in-hospital mortality.

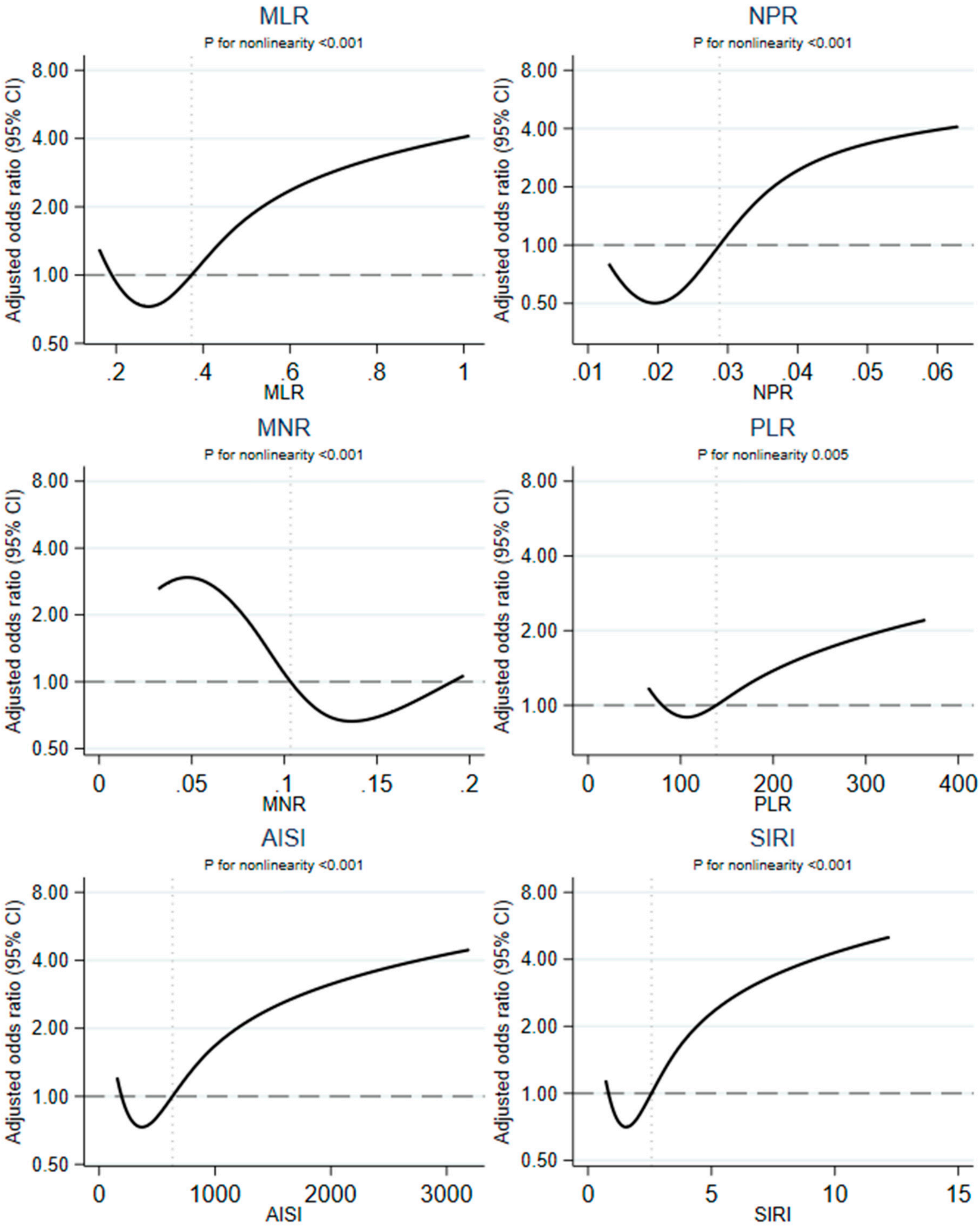

Note: Curves represent adjusted odds ratios for MLR, NPR, MNR, PLR, AISI, and SIRI, with shaded areas indicating 95% confidence intervals. Models were adjusted for age, sex, acute myocardial infarction subtype, serum creatinine, log-transformed cardiac troponin, and diabetes mellitus. The median value of each log-transformed index was used as the reference (OR = 1). Curves are displayed from the 5th to the 95th percentile of each index distribution.
